# Pain Management and COVID-19: A Latin American Perspective

**DOI:** 10.7759/cureus.23100

**Published:** 2022-03-12

**Authors:** Marixa Guerrero, Pablo Castroman, Ovelio Quiroga, Maria Berenguel Cook, Marco Antonio Narvaez Tamayo, Lanfranco Venturoni, Joseph Pergolizzi Jr, Martina Rekatsina, Giustino Varrassi

**Affiliations:** 1 Pain Management, Clinica la Colina, Bogota, COL; 2 Department of Anaesthesiology, University of Montevideo, Montevideo, URY; 3 Pain Medicine, Clinica del Dolor, Coquimbo, CHL; 4 Pain and Palliative Care, Totalcare-Oncosalud-Auna, Lima, PER; 5 Pain Medicine, Hospital Obrero, La Paz, BOL; 6 Internal Medicine, Mazzini Teaching Hospital, Teramo, ITA; 7 Pain Medicine and Critical Care Medicine, NEMA Research, Naples, USA; 8 Pain Management, Basildon University Hospital, London, GBR; 9 Pain Management, Paolo Procacci Foundation, Rome, ITA

**Keywords:** public health, palliative care, covid, pain management, pain

## Abstract

Vaccinations and therapeutics have been developed for COVID-19, but vaccine uptake varies markedly among countries. Public health responses have also varied, in particular, with lockdown efforts and school closing. All over the world, the pandemic exposed healthcare and economic weaknesses. COVID-19 exacerbated mental health issues by exposing the population to prolonged periods of fear, anxiety, financial stress, psychological uncertainties, and sometimes isolation from even family and friends. Chronic pain patients have been disproportionately affected. The pandemic-associated stresses may have exacerbated their already painful symptoms while at the same time interrupting their access to care. The ramifications of the COVID-19 post-viral syndrome (“long COVID-19”) are not yet known. COVID-19 viral infection has been associated with neuropathic pain symptoms. Tele-triage and telehealth applications can help manage chronic pain patients in the COVID-19 era, but many interventional procedures, injections, or other treatments have been delayed. The role of palliative care for patients with terminal cases of infection must be re-examined. Palliative care is a relatively new medical specialty and allows terminally ill patients to die in as much comfort and peace as can be afforded to them. More training in palliative care for all clinicians is urgently needed. COVID-19 exposed much that is wrong or weak or inadequate in our healthcare systems, but it also allowed us to embrace new technologies and develop better systems to manage the challenge of a pandemic.

## Introduction and background

The COVID-19 pandemic exposed the global healthcare system to an unprecedented challenge. Indeed, few people thought of a “global” healthcare system until the pandemic. In Latin America, the first case of COVID-19 was reported in Brazil in February 2020, much later than the first cases in China, the United States, and Europe [1]. Necessary equipment ranging from personal protective equipment (PPE) to ventilators was in short supply. Quarantines, lockdowns, masking, and social distancing meant that healthcare-as-usual was interrupted. On the one hand, new telehealth applications were developed to work around these problems, but, on the other hand, many routines and non-emergency care were halted. This has had a particularly adverse impact on patients dealing with chronic pain. As more is being learned about COVID-19 and its management, new insights and techniques into pain care have been advanced.

## Review

COVID-19 epidemiology in Latin America

The original timeline of the pandemic described patient 0 as a senior man in Wuhan, China, who encountered the novel SARS-CoV-2 virus from a game sold at a wet market [2], but new evidence reports that the first patient may have been infected in China in October 2019 [3]. The origin of the virus has become controversial [4]. The first cluster of patients reported was in China in January 2020, and by January 12, 2020, the genomic sequence of the virus was published. While polymorphisms accumulate in all RNA viruses, specific proofreading functions in SARS-CoV-2 have slowed its replication rate, making mutations take longer than other viruses, such as influenza or norovirus [5]. COVID-19 arrived in Latin America on February 26, 2020, when a 61-year-old Brazilian man returned home from a visit to Italy, which at the time was experiencing a COVID-19 outbreak [6]. As of October 5, 2021, 4.8 million people have died of COVID-19 worldwide, and there have been 236 million confirmed cases in 221 countries and territories [7]. Worldwide, 34.5% of the population is vaccinated, with an estimated 6.36 billion vaccine doses administered to date [8].

The pandemic exposed broad global disparities in healthcare. For instance, only 2.3% of the population of underdeveloped countries have received at least one dose of the vaccine [8]. Seventy-one percent of Brazilians have received one or more doses of the vaccine, much higher than the global average of 46% [8]. Peru and Mexico are about at the mean with 49% vaccination, while Uruguay has 79% of its population vaccinated. South America has an overall vaccination rate of 63% population vaccinated with at least one dose [8]. Latin America has been particularly hard hit by the pandemic, with an estimated 90 million people infected. Accurate statistics can be challenging to obtain and verify because the pandemic is a real-time event, moves quickly, and testing capacity as well as the ability of various countries to gather statistical data varies. The so-called “third wave” of COVID-19 has had a devastating impact on parts of Latin America and Mexico [9].

Latin American nations responded in different ways. For example, lockdowns in some Latin American nations were lax, late, or incomplete. However, Peru, a particularly hard-hit nation, had one of the first lockdowns in Latin America, and it was strictly enforced [10]. Some Latin American nations did not prioritize senior citizens for the vaccine, exposing a vulnerable subpopulation to an elevated risk for infection. Efforts to make people aware of the benefits of masking were sometimes haphazard. Some nations in Latin America, such as Mexico, shared a similar problem as the United States, in that public health is decentralized to the point that centralized efforts to curb infections could not always be implemented [9].

The highest mortality rate for COVID-19 in Latin America occurred in Peru with 606.41 per 100,000 inhabitants, followed by Brazil (268.06), Colombia (244.25), and Argentina (241.19) [11]. However, Latin America is a large and heterogeneous region, and the pandemic has resulted in asymmetric crises within Latin America in terms of the healthcare system, the economic system, and social structures. Structural weaknesses were exposed. In general, Latin America does not have a robust hospital infrastructure to manage emergency care on the scale of this pandemic. There were not always sufficient healthcare professionals to manage local caseloads, and many hospitals lacked sufficient critical-care beds to meet the needs of the pandemic. In addition, Latin America faced specific challenges: there was a general distrust of the healthcare system, a disbelief in the seriousness of the pandemic, and a strong interest in traditional medicine and alternative treatments.

The pandemic highlighted an urgent need for the education of healthcare workers and reliable information for patients and the general public. Vaccination efforts have been strongly promoted in Latin America with good results, but the world is now confronting variants, new outbreaks, and postviral complications, all of which have added new burdens [12,13]. Anxiety, illness, financial insecurity, and the pressures of prolonged lockdowns have created stressful situations for the general population. The COVID-19 pandemic also showed that many hospitals around the world, including those in Latin America, need more training to manage advanced diseases, better equipment, more stores of personal protective equipment (PPE), specific protocols for end of life, and better training of healthcare professionals for pain management. Urgent care was also insufficient in many places because people suffering dyspnea, high fevers, respiratory distress, and tachycardia were not always rapidly and appropriately treated.

There is much that can be learned from the pandemic. Greater healthcare literacy is needed. Care in the pandemic should be “de-medicalized” so that different treatment options are available depending on the patient and the trajectory of the illness. We must encourage the population to be autonomous and empowered in managing their risk of infection and its consequences. This means, in part, providing the general population with realistic strategies for treatment, supportive care, accurate but understandable public health information, and encouragement. Virtual care has emerged as an unintended benefit of the COVID-19 pandemic, and patients seem more accepting of telehealth than they were previously. Support groups and networks of patients who can encourage each other should be facilitated to proactively address mental health issues.

There is much that COVID-19 has taught healthcare professionals and the public health system. The pandemic caused global healthcare to face up to systemic vulnerabilities that must be recognized and defined to be corrected. COVID-19 has created a climate of uncertainty that challenged clinicians, but in a crisis, the healthcare system must be able to manage uncertainty. Clinicians faced great adversity during the pandemic as they struggled with difficult caseloads, insufficient supplies and equipment, lack of PPE, uncertain pandemic progression, lack of clear-cut public health guidance, and their own risk of infection. Ideally, these healthcare workers need to build robust personal networks of family and friends and other support structures to see them through periods of crisis. More education is needed in infectious disease, triage, and other aspects of emergency medicine. Finally, healthcare workers around the world must have and maintain high levels of professional empathy, even when they face what can seem like insurmountable challenges. COVID-19 will become endemic and lose its pandemic status in the future, but epidemics and pandemics will recur, and we need to learn these important lessons now to weather the next outbreaks more safely and effectively.

Painful syndromes in COVID-19 

The COVID-19 pandemic has had broad ramifications, adversely impacting people who contracted the infection, healthcare professionals who cared for them, healthy people seeking to avoid infection, and the large population of individuals who struggle daily with chronic pain [2]. Each has faced unique and important challenges in this pandemic. COVID-19 has presented extreme situations for the general population: abrupt sickness and death of loved ones, financial loss, lockdowns, emotional devastation, insecurity, fear of infection, anxiety about the future, separation from family and friends, drastic changes in work and travel, a sense of “disconnectedness,” uncertainty about public health data and statistics, and other psychosocial stresses which will likely extend into the future [14]. The pandemic exposed the general public and the healthcare profession to an avalanche of sometimes contradictory information, which in some cases made people mistrustful [15].

Chronic pain patients have been disproportionately impacted by the pandemic [16]. Chronic pain has been described as a multidimensional biopsychosocial phenomenon involving the complex interplay of biological, psychological, and social factors [17]. Thus, the psychological and social stresses of a prolonged pandemic could increase the prevalence of chronic pain and exacerbate painful symptoms. Patients already dealing with chronic pain now suddenly were confronted with personal and financial loss, isolation, new medical paradigms for obtaining care, in some cases denial or delay of care, and the day-to-day fear of contracting the infection.

Long-haul COVID-19 or long COVID-19 is a postviral syndrome that was first reported online by people discussing their symptoms after COVID-19 resolution and has since been described in the literature [18]. Symptoms of long COVID-19 are many, but prominent among them are chronic fatigue, diffuse myalgia, and sleep disorders [19]. Postviral syndromes have occurred with other viruses, such as the severe acute respiratory syndrome (SARS) epidemics in 2002 [20]. Gastrointestinal infections are strongly linked to postviral syndromes in general, and about 10% of patients who develop certain enteric infections will develop irritable bowel syndrome [21]. The exact trajectory of long COVID-19 remains unknown, but it appears that risk factors for prolonged symptoms include the severity of the acute COVID-19 infection, its duration, and the premorbid psychological status of the patient [22]. Although older age is a risk factor for acute COVID-19 infection, it does not seem to be a risk factor for long COVID-19 [22]. Moreover, the symptoms that occurred in acute COVID-19 are not necessarily the same as the symptoms that emerge in long COVID-19 [19].

In the aftermath of the apex of the pandemic, there emerged three distinct new subpopulations of chronic pain patients. The first are those whose chronic pain first emerged after COVID-19 infection and might be considered a form of postviral chronic pain; the pain relates either directly to long COVID-19 or to organ damage that occurred during the acute infection [13]. The second subpopulation includes those who had pre-existing chronic pain that worsened after the COVID-19 infection. The third emerging chronic pain subpopulation is less well studied and consists of individuals who did not have chronic pain before acute infection but who developed chronic pain during the pandemic. These individuals had psychological and psychosocial risk factors that predisposed them to chronic pain, such as anxiety, catastrophizing, fearfulness, loneliness, depression, and the stresses of the pandemic triggered painful symptoms. In addition to these three new subpopulations, there is a broad population of people with pre-existing chronic pain whose pain continued but did not worsen or change throughout the pandemic.

In this connection, it is important to recognize that much intensive care unit (ICU) patients develop “post-intensive-care syndrome” that can be associated with chronic pain [23]. ICU hospitalization for any cause may result in cognitive dysfunction, weakness, and intrusive thought patterns similar to post-traumatic stress disorder that may persist long after ICU discharge [24]. Of ICU patients on mechanical ventilation, 56% report some form of impairment after critical illness enduring for at least a year [25]. Among survivors of the earlier severe acute respiratory syndrome (SARS) epidemic in Hong Kong, 25% to 44% suffered post-traumatic stress disorder, and 15% had depression after the infection [26].

The symptoms of long COVID-19 include limb and joint pain, headache, neuralgia, myalgia, abdominal pain, chest pain, angina, and others [27]. Among COVID-19 survivors, many will experience chronic pain with a neuropathic component after the infection [28]. Different body systems can be affected, such as the respiratory, cardiovascular, hepatic, and other systems [27]. The distinction between pain in long COVID-19 and new-onset chronic pain is not clear because it may be possible for certain painful conditions in long COVID-19 to transition to chronic pain. Patients who survived COVID-19 have a significantly higher incidence of de novo chronic pain than patients who were not infected (65.2% vs. 11.0%, respectively, p=0.001) and a higher prevalence of de novo headaches (39.1% vs. 2.7%, p=0.001) [29]. While acute and long COVID-19 symptoms are not always correlated, headache at the onset of acute COVID-19 infection has been associated with long-term headache and fatigue that persisted up to seven months after hospital discharge [30]. When headache occurs in acute COVID-19 infection, the patient should be monitored for persistent headache following the infection.

Among COVID-19 patients discharged from the hospital, 27.3% reported persistent joint pain [31]. In a study of 41 hospitalized COVID-19 patients in China, 44% had myalgia, of whom 33% had elevated levels of creatine-kinase [32]. In a case series of 99 patients with diagnosed COVID-19, 11% of patients reported myalgia, of whom 13% had elevated creatine-kinase levels [33]. The SARS-CoV-2 virus enters the body by way of the interaction of its spike domain with the host’s angiotensin-converting enzyme (ACE)-2 receptor. ACE-2 receptors are dense in the musculoskeletal system, and it has been speculated that the virus may directly impact skeletal muscle [34]. The body’s systemic inflammatory response during infection may also play a role in myalgia [34].

Perhaps most important in considering chronic pain after COVID-19 is the role of neuropathic pain, which may manifest as Guillain-Barre syndrome, myopathy, polyneuropathy, peripheral nerve injury, and other forms of peripheral or central neuropathy [28]. Neuropathic pain has been reported after Zika, chikungunya, herpes zoster, HIV, and other viruses [28]. However, neuropathic pain has been associated with ICU hospitalization for any cause. Neuropathic pain following ICU hospitalization has been attributed to prolonged time spent in a prone position, lack of mobility, neuromuscular blocks, muscle atrophy, complications due to procedures such as chest tubes or tracheotomy, critical illness myopathy, and polyneuropathy [35]. (See Table 1)

**Table 1:** Risk Factors for Chronic Pain Among Hospitalized COVID-19 Patients

Domain	Challenges
Rehabilitation challenges	Potential for overburdened rehabilitation services. Lack of coordinated rehabilitation pathways. Risk of second and future waves diverting resources. Lack of specific COVID-19-related rehabilitation evidence. Multimorbidities. Fatigue.
Mental health burdens	Risk of posttraumatic stress disorder. Social isolation during admission and post-discharge. Pandemic-specific psychological burdens.
Neurological insult	Neuroimmune response to infection. Risk of neurotropism. Painful neurological sequelae, e.g., stroke.
Risks associated with the intensive care unit	Prolonged ventilation. Prolonged immobility. Neuromuscular block. Repeated proning. Risk of sepsis. Risk of procedural pain.
COVID-19-patient-related factors	High prevalence of comorbidities. Older population.
High risk of acute pain	Painful symptoms of acute infection. Risk of procedural pain. Stretched healthcare workforce. Low priority is given to symptom management.

Chronic neuropathic pain may occur in up to 2.3% of hospitalized COVID-19 patients, although the mechanisms remain to be elucidated [36]. Stroke, myelitis, or Guillain-Barré syndrome, are prevalent among COVID-19 patients and associated with an elevated risk for neuropathic pain [36].

Patients who entered the era of COVID-19 with chronic painful conditions may have suffered worsening symptoms. The lockdowns and overtaxed healthcare systems meant that chronic pain patients could not always get the medical treatments on which they had come to depend. Unemployment, financial distress, overburdened hospitals and clinics, and lapses in insurance coverage also limited care for chronic pain. The stress, anxiety, and loneliness imposed by the pandemic may also have worsened painful symptoms. Finally, during the lockdown, many people, not just chronic pain patients, were unable to get their usual exercise or pursue healthful outdoor activities as normal. These things set the stage for an exacerbation of pre-existing chronic pain. These same factors may have triggered de novo chronic painful conditions in others, likely attributable to fear, anxiety, depression, poor sleep, and forced inactivity. The extreme stress associated with the pandemic affected some people to the extreme, possibly even exacerbating substance use disorder [37,38] or leading to suicidality [39,40].

COVID-19 will have far-reaching implications for pain medicine, and there are urgent and unmet medical needs to help treat the pain-related symptoms of acute and long COVID-19. Dedicated multidisciplinary COVID-19 clinics and registries of infected patients may be helpful to better understand how COVID-19 has affected chronic pain patients. The role of telehealth is a positive consequence of the pandemic and may serve pain patients in the future as a new and convenient way to deliver certain types of healthcare. Above all, it must be recognized that de novo chronic pain or worsening pre-existing chronic pain is prevalent in the pandemic, and prompt, targeted treatment is needed [41].

Pain management in COVID-19

People with pre-existing chronic pain, regardless of their COVID-19 status, may have suffered worsening symptoms as a result of the psychological stresses and social limitations associated with prolonged isolation [41]. Many chronic pain patients dependent on the healthcare system and their caregivers may have felt abandoned during lockdowns if their routine care had to be interrupted [42]. Chronic pain patients who contracted COVID-19 ideally require a nuanced approach to care that both controls pain and manages viral symptoms; such care may not have been available or accessible.

Tele-triage describes a telehealth practice that allows a remote clinician to assess patient symptoms, measure the body temperature, assess pain, and categorize the patient. The triage levels in this system should be low for chronic pain patients who have controlled pain and do not have COVID-19; elevated when it is suspected such patients may have COVID-19; and highest when it is suspected that emergency care may be appropriate [43]. Murphy and Latif recommend that triage levels be described as elective, urgent-elective, and urgent [44].

Interventional pain patients may not have received their routine treatments during the pandemic, and a triage system for interventional procedures has been proposed. For example, urgent triage levels are appropriate for chronic pain patients experiencing a worsening of complex regional pain syndrome or degenerative or neurological pain resulting in impaired mobility, while emergency triage levels included pain or conditions that impaired the patient’s ability to conduct the activities of daily living. The lowest triage level, elective, is reserved for conditions that are stable and can be safely and effectively managed remotely, using alternative treatments rather than an interventional procedure [45].

Many interventional pain patients require ultrasound-guided treatments, and during the pandemic, this posed a problem even for those who could get to a hospital or clinic for pain treatment. The sonographer or sonologist must be near the patient during imaging, which requires strict protective measures. The SARS-CoV-2 virus may survive up to nine days on ultrasound equipment and can exist in inert form for up to 96 hours. Critical devices such as ultrasound equipment must be cleaned carefully between patients using a high-grade disinfectant [46]. Ultrasound personnel should use adequate PPE during scanning. Some clinics might not have offered ultrasound during various phases of the pandemic or that patients were too fearful to undergo treatment.

Since perioperative COVID-19 infection increases postoperative morbidity and mortality, patients diagnosed with COVID-19 are asked to delay surgery, if possible, for at least seven weeks until the infection has completely resolved. Those who have symptoms after seven weeks may require further delay [47]. This meant that important procedures to relieve pain had to be postponed for many patients.

The use of analgesics during the pandemic has generated considerable controversy and confusion. Nonsteroidal anti-inflammatory drugs (NSAIDs) may be appropriate for pain control in certain patients, but there remain some unknowns in their use with COVID-19 patients [48]. At first, there was controversy about the use of ibuprofen, often chosen for its antipyretic properties, but these concerns were shown to be unfounded [49]. It was further speculated that NSAIDs may be safe but could potentially mask symptoms of the infection that would be better to recognize early. Pain patients and others may require information about the use of over-the-counter pain relievers such as paracetamol (acetaminophen) or NSAIDs, as there are risks to their use, particularly over a prolonged period.

Opioids are effective analgesics and may be appropriate for treating chronic pain in certain patients, but since opioids can suppress the immune system, their use must be scrutinized and carefully considered in a pandemic [50]. Opioid therapy may also mask symptoms of acute infection, which could delay COVID-19 treatment. Transdermal opioid delivery systems should be used with caution, as heat, even from a fever, can increase drug release or otherwise negatively affect treatment [51]. Patients with prolonged exposure to opioid analgesics may develop withdrawal symptoms if opioids are tapered too quickly or discontinued abruptly. In some cases, telemedicine may be used to provide advice to patients who may need to taper their dose [52,53].

Chronic pain patients may take antidepressants, benzodiazepines, and other adjuvant agents that could potentially result in drug-drug interactions with certain COVID-19 treatments. Antidepressants may interact with hydroxychloroquine, chloroquine, and azithromycin, which may elevate the risk for QT-segment prolongation. Lithium is contraindicated for concurrent use with hydroxychloroquine, chloroquine, and azithromycin. Anticonvulsants and benzodiazepines present little or no risk for adverse interaction with COVID-19 treatments [54].

The use of corticosteroid injections during COVID-19 is controversial. There is some evidence that one intra-articular corticosteroid injection increases the risk of contracting influenza; no study has been done specifically on COVID-19, but this finding is suggestive [55]. No studies have been conducted as to whether a corticosteroid injection might affect the clinical course of COVID-19. If a patient might benefit from a corticosteroid injection during the pandemic or if the patient has acute COVID-19, it is important to exercise clinical prudence in appropriate patient selection, explaining the potential risks to the patient, and obtaining informed consent. If a corticosteroid is to be used, it should be employed at the lowest effective dose for the briefest effective course of treatment [55]. It is not clear if corticosteroid injections might affect the efficacy of the messenger ribonucleic acid (mRNA) vaccine for COVID-19. Based on the understanding that both epidural and intra-articular corticosteroid injections suppress the hypothalamic-pituitary-adrenal axis, out of an abundance of caution, it has been suggested that a corticosteroid injection be administered at least two weeks before an mRNA vaccine or no fewer than one week after [56].

The disruptive impact of COVID-19 on the world has not been entirely negative. Telemedicine, which met with some resistance before the pandemic, has expanded and improved to the extent that it is much more helpful and accepted today than it was just a few years ago. For example, the literature describes a virtual spinal examination, which allows the patient to actively participate in a remote spinal examination that can guide the treatment of back problems [57]. COVID-19 has likely accelerated such innovative telehealth approaches.

The pandemic has created several serious challenges in the treatment of pain. Healthcare professionals must exercise caution and mitigate risk when patients and clinicians are in close contact. Pain patients have faced unprecedented challenges in getting care due to clinic lockdowns, fear of infection, and social distancing. The medical profession must recognize that the pandemic has affected the general population not just physically but also in terms of mental health, and pain patients may not be resilient but instead vulnerable to depression, anxiety, and stress. Healthcare systems can be stretched to the breaking point, forcing clinicians to sometimes make difficult decisions in terms of where and how to direct their efforts [58]. Pain specialists must consider all of these factors and seek to strike a balance in offering responsible care for their patients, protecting themselves, and promoting trustworthy public health policies.

Palliative care

Epidemics and even pandemics have always been with us. COVID-19 is a well-documented and global pandemic, but it does not surpass the plague of Justinian in 541-549 AD in virulence and devastation. This plague, which affected the Roman Empire all of the ways to its eastern capital in Constantinople, was responsible for an estimated 50 million deaths. The plague of Justinian was just the first wave of what would later be known as the Black Plague, which waxed and waned over the centuries. The worst outbreak occurred between 1346-1353 AD in Europe when it is estimated that up to 200 million souls perished. At that time, neither the cause nor the treatment for the plague was known, heightening the fear and terror of the time. Even today, the humanitarian crisis of a pandemic strains our social systems, challenges medicine, collapses the economy, and overwhelms our public health efforts to care for sick and dying individuals. These plagues divert considerable economic and human resources away from the common good and toward local and national solutions aimed only at mitigating suffering, providing whatever incomplete relief is possible. Even when costly, these solutions offer only partial remedies. In the modern world, pandemics should be viewed as humanitarian crises that demand a coherent, coordinated, and global response.

During these crises, palliation emerges as an almost alien concept that contradicts a fundamental notion of modern medicine, namely that patients must be treated as aggressively as possible in the struggle against death. To some extent, the healthcare systems view death as defeat and illness as inherently manageable, even if it cannot always be cured. COVID-19 has shown our healthcare systems that we must think seriously about the role of palliative care.

The first case of COVID-19 in Peru occurred on March 5, 2020, when an infected traveller arrived from Europe. Since that day up to July 28, 2021, there have been 2,108,595 confirmed cases of COVID-19 in Peru; 13,348,711 negative COVID-19 tests; and 196,214 COVID-19 deaths. The Peruvian Minister of Health used existing laws to modify the nation’s current telemedicine system into the Teleatiendo, which allows for patients to be monitored, treated, and attended by phone or instant messaging service throughout their illness [67]. Further legal modifications were quickly implemented to allow for opioids to be prescribed by phone. In these ways, Peru was a leader in adapting healthcare in innovative ways to meet the challenge of the pandemic [59]. However, this raised an important and ethically delicate issue: certain patients were not going to survive COVID-19. These patients were offered palliative care, allowing the healthcare system to prioritize scarce resources toward those patients with a better likelihood of survival but without neglecting the needs of patients nearing the end of life. Palliative care alleviates pain and suffering and does not facilitate death. Instead, palliative care recognizes the inevitability of death for certain patients and seeks to provide them comfort, dignity, spiritual support, and symptomatic care in their final days [60].

The concepts of palliative care are relatively new, and even healthcare professionals may find them off-putting. Training is urgently needed to raise awareness about the value of palliative care and its main principles. In palliation, an individual patient assessment must be made regarding the disease, its severity, and its prognosis, taking into account individual patient factors, such as comorbidities, frailty, and patient age. If the patient’s situation is severe, not likely to change, and would not benefit from treatment (or if treatment would cause more suffering than benefit), the goal shifts from treatment to alleviation of suffering. The patient is not abandoned, but he or she is not subjected to extreme and possibly painful efforts to delay death through aggressive treatments.

A core goal of palliation is providing the patient comfort and peace as life comes to an end, which has particular relevance in the COVID-19 pandemic [61]. The isolation imposed by lockdowns, the strict protocols enforced by hospitals, and the fearfulness in society may serve to heighten the alienation, suffering, and pain in the dying patient. As much as possible, palliative care patients must be allowed to say goodbye to family and friends and to take what small comforts they can find in familiar settings. Palliative care patients should be given medications to ease their pain and relieve their emotional turmoil. The goal of palliation is to allow as much peace and dignity for the patient and their loved ones as possible as death approaches [62].

Palliative care training is essential for healthcare professionals who must work in epidemics, pandemics, or disasters. The fundamental principles include triage, effective but compassionate communications, pain assessment, and alleviation of suffering [63]. This must overcome modern clinical training that emphasizes the “hero mentality” where aggressive and even risky treatments may be used to prolong life at all costs. It is not a medical failure to recognize that some patients will not survive, and these patients require a different kind of care. Instead, palliative care makes the distinction between those who can fight and survive versus those who should be comforted as they pass away.

Pandemics, epidemics, outbreaks of virulent infectious diseases, and other disasters will undoubtedly recur. They deserve more study to increase global and local preparedness and find optimal strategies to take decisive, effective actions as soon as possible. At the same time, the concept of palliation must not be jettisoned. Governmental, non-governmental, and other official agencies must be transparent and forthright when sharing information. The climate of fear around COVID-19 was in part due to confusion among people who were unsure about the risks of the disease or how to protect themselves. Better and more direct communication is essential.

A centralized crisis management system may be helpful to create a “defense system” of epidemiologists, public health experts, healthcare professionals, and government leaders who can respond quickly to disease outbreaks [64]. In new outbreaks, priority should be given to developing reliable diagnostic tools, effective treatments, and vaccines, if appropriate. PPE and other specialized equipment must be stockpiled for rapid deployment where needed. Emergency stores should be set up so that even under-developed and poor nations have ready access to PPE and other equipment. This process can be formalized so that local governments may request from a central authority the basic necessities, medicines, PPE, and other necessary tools to fight disease when an outbreak is declared. This sort of system could distribute supplies where needed fairly and transparently.

Peru has implemented and expanded several telemedicine centers that treat not only COVID-19 but also oncology patients and others at high risk and/or with complex conditions during the pandemic [65]. In Peru, 2,267 new cancer patients were treated with telemedicine in 2016; this number increased to 2,472 in 2018 [65]. In January and February, as well as July and August of 2020, Peru saw the highest number of telemedicine consultations, which mirrored pandemic activity [65]. While there is considerable interpatient variation, the approximate average time for a telemedicine consultation is about 45 or 60 minutes for an existing or new patient, respectively.

Rapid and effective response to a healthcare emergency is possible if a trained team and an established protocol are already in place. In Peru, we could provide care for virtually all of the population infected with COVID-19 during the pandemic. Remote regions of Peru, like remote regions in all parts of the world, were hard hit [66]. Systems must proactively find solutions to send resources quickly to remote regions. These response efforts must also recognize that palliative care is an essential component of emergency healthcare. In Peru, it was possible to provide palliation to many appropriate patients using telemedicine models and healthcare professionals trained in palliative care. What is needed in Peru and around the world is an influx of healthcare professionals better trained for emergency and palliative care. The COVID-19 pandemic is not over. It is imperative for us as medical authorities and healthcare professionals to acknowledge that the pandemic may worsen, may resolve but recur, or those new variants may arise.

## Conclusions

In Latin America, as in the rest of the world, COVID-19 exposed numerous vulnerabilities in our healthcare system as hospitals were strained to accommodate burgeoning caseloads. Latin America faced specific challenges in that there was a cultural distrust of the healthcare system, a skepticism about the seriousness of the pandemic, and a strong interest in alternative medicine. However, vaccination uptake has been generally strong in Latin America. COVID-19 is associated with certain painful symptoms, such as myalgia and headache, and post-viral syndrome or “long COVID-19” has been linked with a range of symptoms, including neuropathic pain. Patients with pre-existing chronic pain before the pandemic may have had their pain care routines interrupted, treatments delayed or cancelled, and symptoms exacerbated by lockdowns, social isolation, anxiety about the disease, and financial setbacks. Triage systems have been developed to prioritize pain care in COVID-19, which is complicated by the risk of disease transmission. Ultrasound guidance, for example, used in certain interventional pain treatments, was sometimes off-limits during COVID-19 for fear of disease transmission. While COVID-19 has been devastating on many fronts, it also brought with it advances and greater acceptance of telemedicine and greater awareness of the need for palliative care for patients at the end of life.
